# A review on the immunomodulatory activity of *Acanthopanax senticosus* and its active components

**DOI:** 10.1186/s13020-019-0250-0

**Published:** 2019-07-31

**Authors:** Kit-Man Lau, Grace Gar-Lee Yue, Yuk-Yu Chan, Hin-Fai Kwok, Si Gao, Chun-Wai Wong, Clara Bik-San Lau

**Affiliations:** 1 Institute of Chinese Medicine, The Chinese University of Hong Kong, Shatin, New Territories Hong Kong; 2 State Key Laboratory of Research on Bioactivities and Clinical Applications of Medicinal Plants, The Chinese University of Hong Kong, Shatin, New Territories Hong Kong; 3 Li Dak Sum Yip Yio Chin R & D Centre for Chinese Medicine, The Chinese University of Hong Kong, Shatin, New Territories Hong Kong

**Keywords:** *Acanthopanax senticosus*, Acanthopanacis Senticosi Radix et Rhizoma seu Caulis, Chinese medicine, Immunomodulation, Isofraxidin, Syringin, Eleutheroside E

## Abstract

Acanthopanacis Senticosi Radix et Rhizoma seu Caulis, the dried root and rhizome or stem of *Acanthopanax senticosus*, is commonly known as Siberian ginseng or Ciwujia in Chinese. It is used all over the world as an adaptogen to enhance physical and mental performance for the sake of normal physiological functioning of human bodies under stress. In the theory of traditional Chinese medicine, Ciwujia can strengthen the spleen that is an essential organ for immunological response. Its traditional applications include inflammation, fatigue and cancer in which the immune-regulating function is always involved. In this article, the immunomodulatory activities of Ciwujia extracts, fractions and pure compounds were extensively reviewed first. Then, the possibility of upgrading the chemical markers to bioactive markers was explored. Finally, the potency of aqueous extract and ethanol extract in regulating cytokines production from human peripheral blood mononuclear cells was compared. We conclude that although various phytochemicals such as isofraxidin, syringin and eleutheroside E from Ciwujia have been shown to modulate immunological functions, the aqueous extract of Ciwujia as a whole possesses the most potent efficacy. Therefore, aqueous (rather than ethanol) extract of Ciwujia should be used in order to benefit from its immunomodulatory properties.

**Electronic supplementary material:**

The online version of this article (10.1186/s13020-019-0250-0) contains supplementary material, which is available to authorized users.

## Background

Acanthopanacis Senticosi Radix et Rhizoma seu Caulis, commonly known as Siberian ginseng or Ciwujia in Chinese, is the dried root and rhizome or stem of *Acanthopanax senticosus* (previously classified as *Eleutherococcus senticosus*). According to Chinese Pharmacopoeia, it is a traditional Chinese medicine widely prescribed to nourish qi, fortify the spleen, tonify the kidney and tranquilize the mind [1]. It is also a functional food renowned for its anti-fatigue [2, 3], neuroprotective [4, 5] and immunomodulatory activities [6–11]. In this review, the immunomodulatory action and the corresponding active components of Ciwujia will be discussed. Moreover, attempt was made to compare the potency of aqueous extract with that of ethanol extract in regulating cytokines production from human peripheral blood mononuclear cells so as to provide more insights on how Ciwujia should be prepared in order to benefit from its immunomodulatory properties.

The immunomodulatory effect of Ciwujia was clinically proven in the 1980s. A double-blind placebo-controlled study conducted by Bohn and colleagues demonstrated that ethanol Ciwujia preparation caused a drastic increase in the absolute number of immunocompetent cells, with an especially pronounced effect on T lymphocytes, predominantly of the helper/inducer type, and also on cytotoxic and natural killer cells of healthy volunteers [6]. Thereafter, a number of scientific research works were conducted to elucidate its action in more details.

### Aqueous extract and ethanol extract

Both aqueous extract and ethanol extract of Ciwujia have been reported for their immunological effects.

Oral administration of aqueous extracts of Ciwujia samples from five different sources at 1 g/kg body weight for consecutive 9 days could significantly prolong the swimming time of male C57 BL/6J mice. Some of the aqueous extracts inhibited the reduction of natural killer (NK) activity in forced swimming stressed mice [7]. The result of NK-stimulatory effect was in line with that reported by Yoon’s team [8]. Intravenous administration of GF100 (an aqueous infusion of Ciwujia) significantly augmented NK cytotoxicity of BALB/c mice towards murine T cell lymphoma Yac-1 cells. On the other hand, GF100 significantly inhibited lung metastasis of colon 26-M3.1 cells in a dose-dependent manner and this effect was completely abolished if NK cells were depleted by injection of rabbit anti-asialo GM1 serum. Besides NK cells, splenocytes and macrophages were also the immunological targets of GF100. Treatment with GF100 enhanced the proliferation of concanavalin A (Con A)-stimulated splenocytes dose-dependently. Moreover, treatment of peritoneal macrophages with GF100 in an in vitro experiment induced the production of various cytokines such as IL-1β, TNF-α, IL-12 and IFN-γ in a dose-dependent manner. In addition, peritoneal macrophages obtained from GF100-treated mice displayed a higher cytolytic activity against tumor cells than those from the untreated mice. Therefore, it was concluded that the antitumor effect of GF100 was associated with activation of macrophages and NK cells [8]. Wang and colleagues also reported that an aqueous preparation of Ciwujia could significantly increase the phagocytic function of monocytes in mice [9].

Ethanol extract of Ciwujia also exerted immunomodulatory activity. A pharmaceutical Ciwujia ethanol preparation was found to influence markedly the chemokine and cytokine syntheses from activated whole blood cultures of healthy volunteers. It increased the release of Rantes but decreased the production of IL-4, IL-5 and IL-12. Depending on the concentration used, its effect on G-CSF, IL-6 and IL-13 could be either stimulatory or inhibitory [10]. Another 40% aqueous ethanol extract (AE) has been reported to be able to counteract the toxic effect of cadmium that induced changes in the immunoregulatory mechanisms of a host. Oral administration of AE for 8 weeks led to a significant decrease in cadmium levels and increased the amount of macrophages and both B and T lymphocytes in spleens of cadmium chloride-intoxicated mice [11].

### Polysaccharides and glycoproteins

Polysaccharides and glycoproteins are two active immunomodulatory fractions of Ciwujia.

Ciwujia polysaccharides (PES), when injected intraperitoneally at 100 mg/kg for 4–6 consecutive days, could significantly increase the count of IgM antibody plaque-forming cells in mice challenged with sheep red blood cells, and also significantly increased the anti-BSA antibodies and greatly enhanced phagocytosis of peritoneal macrophages [12]. Also, PES itself showed strong mitogenic activity on mouse spleen cells in a dose-dependent manner and it could augment the stimulatory activity of lipopolysaccharide (LPS) on lymphocyte transformations [12].

More recently, Han’s team investigated the mechanism of the immunomodulatory action of a polysaccharide fraction isolated from a cell culture of *A. senticosus* (ASP) using in vitro assays [13]. They found that ASP increased the proliferation of B cells and increased the polyclonal IgM antibody production of B cells dose-dependently. Moreover, ASP stimulated murine peritoneal macrophages, as reflected by the increase in the mRNA and protein expressions of such cytokines as IL-1β, IL-6 and TNF-α. Furthermore, ASP increased the expression of the inducible nitric oxide synthase (iNOS) gene and the production of NO. Based on the finding that the activities of ASP on B cells and macrophages were significantly reduced by treating the cells with antibodies to Toll-like receptor (TLR)-4 and TLR-2 prior to ASP, it was concluded that ASP activates B cells and macrophages via TLR signaling pathway. However, it was shown that ASP did not activate T cells [13].

*Acanthopanax senticosus* polysaccharides work not only in in vitro systems or on normal rodents, but also on immunocompromised/immunologically challenged animals. An acidic polysaccharide fraction was reported to increase the thymus and spleen indexes, increase leukocytes count in the peripheral blood, enhance phagocytic function of macrophages, increase TNF-α, IFN-γ and serum hemolysin levels, enhance splenocyte proliferation, and decrease splenic lymphocyte apoptosis rate in cyclophosphamide-induced immunosuppressed mice [14]. In China, *A. senticosus* is commonly used as a dietary supplement by veterinarians to promote animal health. It could enhance the cellular and humoral immune responses of weaned piglets by regulating the production of lymphocytes, cytokines and antibodies [15]. On the other hand, dietary *A. senticosus* polysaccharide (ASPS) modulated the release of pro-inflammatory cytokines during immunological challenge. It decreased the elevation of plasma levels and spleen mRNA expressions of IL-1β, IL-6 and TNF-α induced by LPS challenge in piglets [16].

GF-AS was a soluble protein layer fractionated from *A. senticosus* stem bark. It induced cytokine production of peritoneal macrophages and exerted prophylactic effect against lung metastasis induced by colon26-M3.1 tumor cells via activation of NK cells in mice. Using gel chromatography and splenocyte proliferation assay, a glycoprotein named EN-SP was isolated and identified as the active component. EN-SP was about 30.5 kDa and mainly composed of carbohydrates. It could significantly increase cell proliferation of murine splenocytes without mitogenic stimuli and possessed stronger anti-metastatic activity than GF-AS [17].

### Small molecules

Apart from polysaccharides and glycoproteins, some small molecules present in Ciwujia also gain certain attention for their abilities in regulating the immune system.

#### Isofraxidin

Isofraxidin, an active coumarin compound used to authenticate Ciwujia raw herbs [1] or commercial products [18], exerts a broad spectrum of pharmacological effects in various diseases such as osteoarthritis [19], cancer [20, 21], lipid metabolism disorder [22] and Alzheimer’s disease [23].

In the immunomodulatory aspect, isofraxidin mainly exerts anti-inflammatory activity. Its profound effect in ameliorating edema and pain was mediated through the inhibition on LPS-induced production of the pro-inflammatory cytokines, including TNF-α and the phosphorylated mitogen activated protein kinase (MAPK) signaling molecules p38 and ERK1/2, from macrophages [24]. Isofraxidin also suppressed the protein expression of NF-κB, levels of NO and IL-6 in serum and production of TNF-α in liver of LPS-challenged mice [25]. Moreover, it protected mice against acute lung injury via the inhibition of cyclooxygenase-2 (COX-2) protein expression and the reduction of inflammatory cells infiltration into lung tissues [26]. As demonstrated in chondrocytes isolated from osteoarthritis patients, pretreatment with isofraxidin prior to IL-1β could inhibit IL-1β-stimulated expression of iNOS and COX-2 that in turn blocked the production of nitric oxide (NO) and prostaglandin E2 (PGE2). In addition, isofraxidin remarkably inhibited mRNA levels and secretion of matrix metalloproteinases (MMPs). It was concluded that isofraxidin inhibited IL-1β-induced joint inflammation via the regulation of NF-κB signaling [27].

#### Syringin (Eleutheroside B)

Syringin, also named eleutheroside B, belongs to the lignan chemical compound group. It is the chemical marker listed in the 2015 edition of Pharmacopoeia of the People’s Republic of China (CP 2015) for assay of Ciwujia. As stipulated, dried Ciwujia samples should contain not less than 0.05% of syringin in weight [1]. Syringin is one of the main active compounds of Ciwujia and reported to possess anti-diabetic [28, 29], anti-fatigue [30, 31], sleep-potentiating [32], neuroprotective [33] as well as antioxidant [34] activities.

Syringin depicts immunomodulatory rather than immunostimulatory effect. It inhibited in vitro immunohaemolysis of antibody-coated sheep erythrocytes induced by guinea pig serum through suppression of C3-convertase of the classical complement pathway [35]. Apart from humoral immunity, cellular immunity is also involved in syringin-elicited immunological response. Syringin significantly inhibited both TNF-α production from LPS-stimulated murine macrophage RAW 264.7 cells and cytotoxic T cell proliferation in a dose-dependent manner [36]. Recently, Ahmad and colleagues further demonstrated that syringin potently reduced the chemotaxis, phagocytic activity, ROS and NO productions and secretions of IL-1β, TNF-α, IL-6, PGE2 and MCP-1 of activated RAW 264.7 cells [37]. With the observation that syringin significantly suppressed fluorescein-isothiocynate (FITC, a hapten which is able to trigger allergic reactions)-induced ear edema in mice but not the ear edema induced by croton oil or arachidonic acid, it was speculated that syringin exhibited anti-allergic effect but not anti-inflammatory effect [36].

#### Eleutheroside E

Eleutheroside E is another lignan isolated from Ciwujia. It could attenuate anesthetic-induced cognitive dysfunction in aged animals [38] and ameliorate diabetes by enhancing glucose uptake, improving insulin resistance and regulating glucose metabolism in type 2 diabetic mice [39]. Using bioactivity-guided fractionation, it was also found to be the active constituent of Ciwujia for combating fatigue [40].

Eleutheroside E exhibited immunomodulatory effect and acted against collagen-induced arthritis (CIA) by suppressing inflammatory cytokine release. The eleutheroside E-treated CIA mice showed significant lower serum levels of TNF-α, IL-6 and IL-23 than those from vehicle-treated mice. Its immunomodulatory effect was further confirmed in cultured macrophages of which the productions of TNF-α and IL-6 were profoundly suppressed in dose- and time-dependent manners [41]. As reported by Kimura and Sumiyoshi, two out of five tested Ciwujia aqueous extracts could recover the reduction of NK activity in forced swimming stressed mice. HPLC analysis revealed that these two extracts contained the highest amount of eleutheroside E. Therefore, it was speculated that eleutheroside E was the active immunostimulatory component in Ciwujia extract [7].

## Discussion

More than one hundred compounds have been isolated from *A. senticosus* [42]. Among them, isofraxidin, syringin and eleutheroside E are relatively specific to Ciwujia and are usually employed as principal components for quality assessment of Ciwujia extracts and commercial products [18, 43]. Also, isofraxidin and syringin are adopted by CP 2015 as the chemical markers for identification and assay of Ciwujia raw herbs [1]. In order to figure out whether isofraxidin, syringin and/or eleutheroside E play a significant role in the immunomodulatory activity of Ciwujia extract and thus can be used as bioactive marker for biological standardization of Ciwujia, our group has tested the effects of these compounds, in comparison with aqueous and ethanol extracts of Ciwujia, on cytokines production from human peripheral blood mononuclear cells (PBMCs) (for detailed experimental procedures, please refer to Additional file 1 - Experimental methodologies).

**Fig. 1:**
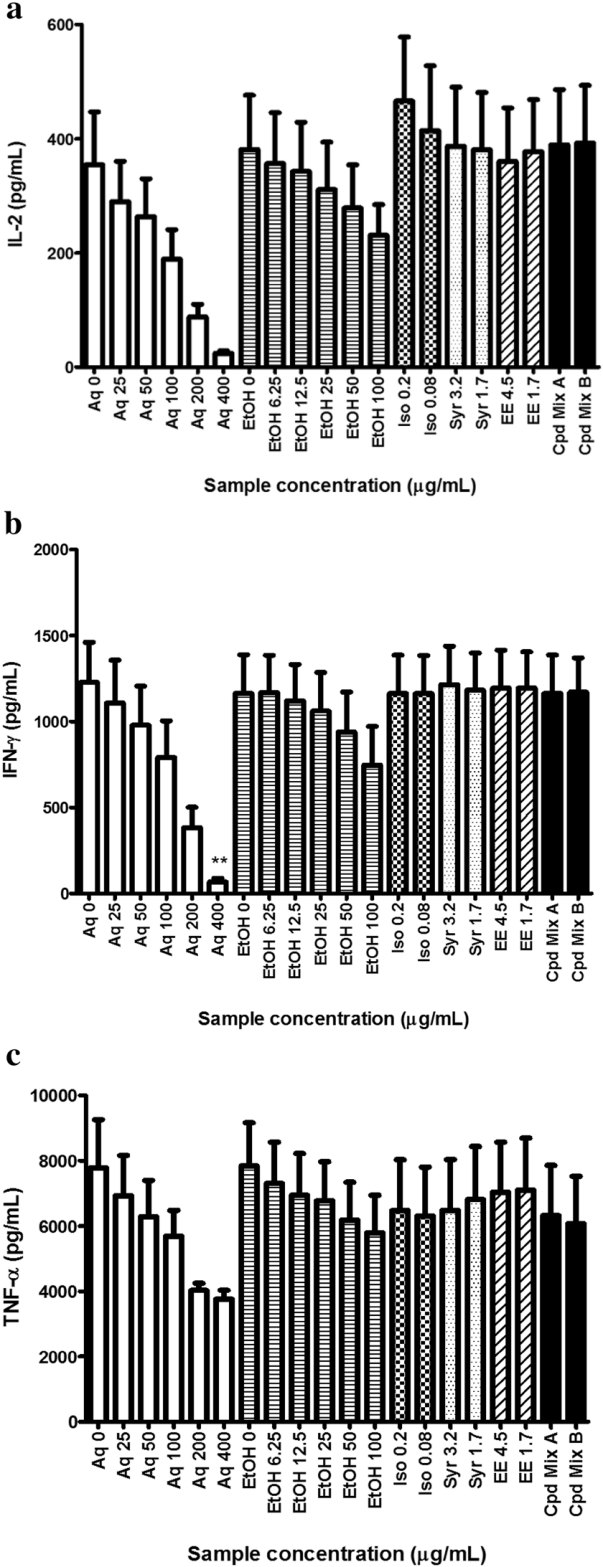
Effect of Ciwujia extracts and compounds on cytokine productions from phytohemagglutinin (PHA)-stimulated human peripheral blood mononuclear cells (PBMCs). PBMCs were incubated with different samples in RPMI-1640 medium plus 10% v/v FBS, 100 units/mL penicillin, and 100 μg/mL streptomycin for 24 h. Culture medium was collected for measurement of cytokine levels using commercial ELISA kits. **a** IL-2; **b** IFN-γ; **c** TNF-α. Data are mean + SEM (n = 5). **p < 0.01 when compared with Aq 0 using one-way ANOVA followed by post hoc Dunnett’s multiple comparison test

As shown in Fig. 1, isofraxidin, syringin and eleutheroside E at corresponding amounts in extracts, either alone or in combination, did not possess any inhibitory effects on phytohemagglutinin (PHA)-stimulated cytokine productions from human PBMCs as the extracts did. It implied that the immunomodulatory efficacies of these three compounds might not be strong enough for them to act as bioactive marker(s) for Ciwujia. Although polysaccharides also possess remarkable immunoregulatory activities as reviewed above, they may not be considered as the potential candidate for bioactive marker because they exist as crude fraction and it is difficult to obtain consistent polysaccharide fraction/pure polysaccharide compounds for quality control purpose of Ciwujia.

On the other hand, Ciwujia aqueous extract (Aq) could dose-dependently inhibit PHA-stimulated productions of IL-2, IFN-γ and TNF-α from PBMCs. Its effect was the most prominent at 400 μg/mL (Fig. 1). Ciwujia ethanol extract (EtOH) also exhibited a trend of inhibition. Although at concentrations below 100 μg/mL, the potency of EtOH was similar to that of Aq, higher concentrations of Aq (without cytotoxicity) could induce greater inhibitory activities in PBMCs. In view of the fact that aqueous extract was more effective than the ethanol extract, Chinese medicine industry should consider using aqueous extract in their proprietary products in order to benefit from its immunomodulatory activities.

## Conclusion

Based on the extensive review and our research findings, we conclude that although certain polysaccharides, glycoproteins and compounds such as isofraxidin, syringin and eleutheroside E from Ciwujia have been shown to potentiate/modulate immunological functions, the aqueous extract of Ciwujia as a whole possesses the most potent efficacies. Therefore, Ciwujia should be prepared as aqueous extract, rather than ethanol extract, for its immunomodulatory properties.

## Additional file

**Additional file 1.** Experimental methodologies.

## Data Availability

Not applicable.

## Abbreviations

Aq: aqueous extract of Ciwujia
CIA: collagen-induced arthritis
Con A: concanavalin A
COX-2: cyclooxygenase-2
CP 2015: 2015 edition of Pharmacopoeia of the People’s Republic of China
EtOH: ethanol extract of Ciwujia
FITC: fluorescein-isothiocynate
iNOS: inducible nitric oxide synthase
LPS: lipopolysaccharide
MMPs: matrix metalloproteinases
NK: natural killer
NO: nitric oxide
PBMCs: peripheral blood mononuclear cells
PGE2: prostaglandin E2
PHA: phytohemagglutinin
ROS: reactive oxygen species
TLR: toll-like receptor
