# Supplementary material for: A review on the immunomodulatory activity of Acanthopanax senticosus and its active components
Source: Chin Med. 2019 Jul 31;14:25. doi: 10.1186/s13020-019-0250-0 (PMC6670126; doi:10.1186/s13020-019-0250-0)
Supplement: Supplementary file 1 — Additional file 1. Experimental methodologies. [file 13020_2019_250_MOESM1_ESM.docx]

**Additional File**

CMED-D-19-00131

A review on the immunomodulatory activity of *Acanthopanax senticosus* and its active components

Lau KM, Yue GGL, Chan YY, Kwok HF, Gao S, Wong ECW, Lau CBS

**S1. Materials and Methods**

*S1.1. Herbal material*

Raw herb of Ciwujia was purchased from renowned supplier in Hong Kong. It was authenticated according to Chinese Pharmacopoeia (CP). Herbarium voucher specimen was deposited in the museum of Institute of Chinese Medicine, The Chinese University of Hong Kong, with the voucher specimen number 3533.

*S1.2. Culture medium, chemicals and reference standards*

Cell culture medium RPMI-1640, fetal bovine serum (FBS), penicillin, streptomycin, and phosphate-buffered saline (PBS) were purchased from Invitrogen (Grand Island, NY, USA). Ficoll-Paque^TM^Plus was obtained from GE Healthcare (Pittsburgh, PA, USA). All other chemicals including phytohemagglutinin (PHA), 3-(4,5-dimethylthiazol- 2-yl)-2,5-diphenyl-tetrazolium bromide (MTT) and trypan blue were purchased from Sigma-Aldrich Co. (St. Louis, MO, USA). The ELISA kits for tumor necrosis factor (TNF)-α, interleukin (IL)-2, and interferon (IFN)-γ were purchased from BD Pharmingen (San Jose, CA, USA). All organic solvents were purchased from Lab-Scan (Gliwice, Poland) and were of analytical grade. The reference standards, isofraxidin, syringin and eleutheroside E, were supplied by National Institute for the Control of Pharmaceutical and Biological Products (Beijing, China).

*S1.3. Human peripheral blood mononuclear cells (PBMCs)*

Fresh human buffy coat preparations obtained from Hong Kong Red Cross Blood Transfusion Service was diluted with PBS at a ratio of 1:1. The diluted buffy coat sample was layered on equal volume of Ficoll-Paque^TM^Plus solution at room temperature and then centrifuged at 800 × *g* for 20 min at 18 °C. The thin white middle PBMC layer was collected and the PBMC were washed with PBS twice and centrifuged at 100 × *g* for 10 min at 18 °C. The supernatant was discarded and the PBMC were resuspended in RPMI-1640 medium plus 10 % v/v FBS, 100 units/ml penicillin, and 100 μg/ml streptomycin. The cell number was counted with a hematocytometer and the viability of the cells was checked by trypan blue exclusion assay. Only the isolated cells with 95 % or above viability were resuspended to the target density for experiments.

*S1.4. Preparation of aqueous and ethanol extracts of Ciwujia*

Raw herb of Ciwujia was extracted in 10-fold (w/v) of distilled water or absolute ethanol under reflux for 1 hour for 3 times. After cooling, the extract solution was filtrated and concentrated under reduced pressure. The aqueous extract was then lyophilized to dry powder and the ethanol extract was dried under vacuum to give a dark brown solid. The extraction yields of aqueous extract and ethanol extract were 5.96 % and 2.44 %, respectively.

*S1.5. Quantification of isofraxidin, syringin and eleutheroside E in Ciwujia extracts*

Stock solutions of syringin, eleutheroside E and isofraxidin were prepared individually in methanol at 1 mg/mL. The three stock solution were mixed and diluted into different concentrations by methanol to give a series of standard solution (10, 8, 6, 4, 2, 1 µg/mL for syringin; 20, 16, 12, 8, 4, 2 µg/mL for eleutheroside E; 4, 3.2, 2.4, 1.6, 0.8, 0.4 µg/mL for isofraxidin). On the other hand, 10 mg of dry extract was dissolved in 5 mL methanol and filtered as the sample solution.

UPLC-QTOF analysis was conducted using an Agilent 1290 UHPLC with 6530 QTOF system (CA, USA). The column used was Agilent ZORBAX Eclipse Plus C18 RRHD, 1.8 µm, 3.0 x 100 mm. The chromatographic separation was conducted at 40 °C under gradient conditions at a flow rate of 0.5 mL/min. The LC profile is as follows: Mobile phase: (A) 0.1 % formic acid in deionized and distilled water, and (B) 0.1 % formic acid in acetonitrile; Gradient: 0-3 min, 12-13.5 % B; 3-8 min, 13.5–45 % B. High purity nitrogen was used as the curtain gas with a flow rate of 10 L/min. The gas temperature was set at 350 °C, and the nebulizer pressure was set at 50 psig. Spectra were recorded in negative ion mode at spray voltage of 4000 V. The mass scan range was between 100-1100 m/z. Data analysis was performed using Agilent MassHunter Workstation Qualitative Analysis Software (CA, USA, version B.06.00). Syringin was determined at 417.1450 m/z [M+FA-H]^-^. Eleutheroside E was determined at 787.2727 m/z [M+FA-H]^-^ and isofraxidin was determined at 221.0474 m/z [M-H]^-^.

*S1.6. Cytokine Production of PBMC*

The isolated PBMC (3 × 10^6^ cells/ ml) were seeded into a 96-well flat-bottom microplates and incubated with various concentrations of Ciwujia extracts or testing compounds **(Table 1).** After the incubation at 37 °C in humidified incubator with 5 % CO_2_ for 24 h, the microplates were centrifuged at 300 × *g* for 10 min to obtain cell-free supernatant. The supernatant was collected and stored at -80 °C until cytokine ELISA experiments. The assay was carried out according to the procedures recommended in the ELISA kit manual.

**Table S1. Testing concentrations of different samples in human PBMCs assay.**

| Samples | Testing concentration(s) | Remarks |
| --- | --- | --- |
| Ciwujia aqueous extract (Aq) | 0, 25, 50, 100, 200 and 400 μg/mL | Non-toxic concentration  range for PBMCs |
| Ciwujia ethanol extract (EtOH) | 0, 6.25, 12.5, 25, 50 and 100 μg/mL | Non-toxic concentration  range for PBMCs |
| Isofraxidin (Iso) | 0.2 μg/mL | Amount in Aq 400 μg/mL |
|  | 0.08 μg/mL | Amount in EtOH 100 μg/mL |
| Syringin (Syr) | 3.2 μg/mL | Amount in Aq 400 μg/mL |
|  | 1.7 μg/mL | Amount in EtOH 100 μg/mL |
| Eleutheroside E (EE) | 4.5 μg/mL | Amount in Aq 400 μg/mL |
|  | 1.7 μg/mL | Amount in EtOH 100 μg/mL |
| Compound mixture A  (Cpd Mix A) | 0.2 μg/mL Iso + 3.2 μg/mL Syr  + 4.5 μg/mL EE | Total amount in Aq 400 μg/mL |
| Compound mixture B  (Cpd Mix B) | 0.08 μg/mL Iso + 1.7 μg/mL Syr  + 1.7 μg/mL EE | Total amount in EtOH 100 μg/mL |
